# A device and an app for the diagnosis and self-management of tinnitus

**DOI:** 10.1515/jib-2022-0004

**Published:** 2022-08-30

**Authors:** Pierpaolo Vittorini, Pablo Chamoso, Fernando De la Prieta

**Affiliations:** Department of Life, Health and Environmental Sciences, University of L’Aquila, 67100 L’Aquila, Italy; IBSAL/BISITE Research Group, University of Salamanca, Calle Espejo 12, Edificio I+D+i, 37007 Salamanca, Spain

**Keywords:** acufenometry, app, audiometry, device, tinnitus

## Abstract

Tinnitus is an annoying ringing in the ears, in varying shades and intensities. Tinnitus can affect a person’s overall health and social well-being (e.g., sleep problems, trouble concentrating, anxiety, depression and inability to work). The diagnostic procedure of tinnitus usually consists of three steps: an audiological examination, psychoacoustic measurement, and a disability evaluation. All steps are performed by physicians, who use specialised hardware/software and administer questionnaires. This paper presents a system, to be used by patients, for the diagnosis and self-management of tinnitus. The system is made up of an app and a device. The app is responsible for executing – through the device – a part of the required audiological and psychoacoustic examinations, as well as administering questionnaires that evaluate disability. The paper reviews the quality of the automated audiometric reporting and the user experience provided by the app. Descriptive and inferential statistics were used to support the findings. The results show that automated reporting is comparable with that of physicians and that user experience was improved by re-designing and re-developing the acufenometry of the app. As for the user experience, two experts in Human-Computer Interaction evaluated the first version of the app: their agreement was good (Cohen’s *K* = 0.639) and the average rating of the app was 1.43/2. Also patients evaluated the app in its initial version: the satisfactory tasks (audiometry and questionnaires) were rated as 4.31/5 and 4.65/5. The unsatisfactory task (acufenometry) was improved and the average rating increased from 2.86/5 to 3.96/5 (*p* = 0.0005). Finally, the general usability of the app was increased from the initial value of 73.6/100 to 85.4/100 (*p* = 0.0003). The strengths of the project are twofold. Firstly, the automated reporting feature, which – to the best of our knowledge – is the first attempt in this area. Secondly, the overall app usability, which was evaluated and improved during its development. In summary, the conclusion drawn from the conducted project is that the system works as expected, and despite some weaknesses, also the replication of the device would not be expensive, and it can be used in different scenarios.

## 1 Introduction

Health informatics can be defined as the application of computer science, engineering and telecommunication to healthcare [1]. Accordingly, it regards the use of methods, applications, systems and devices in all that is concerned with both individuals and public health (e.g., [2–4]). Within such a broad research area, this paper focuses on the management of tinnitus.

Tinnitus refers to annoying ringing, buzzing or hissing sounds in the ears, in varying shades and intensities [5]. The prevalence of tinnitus increases with age and is more pronounced in males than females [6]. The overall health and social well-being of patients that suffer from this condition can be affected in a number of ways, ranging from insomnia, poor concentration and anxiety, to an ongoing depression and inability to work [7, 8]. Furthermore, recent studies also pointed out the role of emotional states and emotion dynamics as factors contributing to how tinnitus leads to distress and disability [9, 10]. The evaluation of a patient with tinnitus usually requires a long process, this is because it entails the collection of anamnestic data, a clinical evaluation, audiological examinations, the compilation of questionnaires and appointments with a psychologist. More concretely, a patient affected by tinnitus is first evaluated by an otorhinolaryngologist or audiologist. Then, a multidisciplinary approach is normally adapted which involves neurologists, dentists, internists, radiologists, psychologists and psychiatrists. Psychologists usually play a very important role in the evaluation, which includes conversations and specific tests that check if tinnitus could be provoked by anxiety, depression or low social well-being in general. Other fundamental tests are pure tone audiometry, impedanciometry, and otoacoustic emissions as well as psychoacoustic measurements of pitch and loudness matching (acufenometry), the determination of the minimum masking level of tinnitus and the sound discomfort threshold.

The paper reports the results of the first phase of a project whose aim is to support patients with tinnitus in (a part of) the diagnostic procedure summarised above, i.e., the audiological examination, psychoacoustic measurement of tinnitus, and disability evaluation. Accordingly, the project developed a system, made up of:

– a simple, affordable, platform-independent device that can be connected to a smartphone/tablet, able to execute (a part of) the audiological and psychoacoustic examinations needed to diagnose tinnitus, and
– a dedicated app that controls the device, automates the examinations and the questionnaires needed to measure the disability induced by tinnitus, with an easy-to-use interface through which the execution of examinations and their reporting could be performed directly by the end-users.

There are apps on the market that are able to perform pure-tone audiometry, several of them use calibrated transducers (the latter are expensive, professional devices developed for audiologists). There are also apps that measure tinnitus and its effects, and others that deliver questionnaires. However, to the best of the authors’ knowledge, and drawing on the available literature, summarised in Section 3, this project is the first attempt to develop an *app and an affordable device specifically developed for patients*, capable of performing audiological and psychoacoustic measurements, automatically reporting on them, and evaluating disability through standardised questionnaires, in an *integrated environment* that enables patients with tinnitus to self-manage their condition. Furthermore, the paper reports several usability studies (both with expert and users) and how the results lead to improvements in the app functionalities.

The usefulness of this project is manifold:

– The app simplifies medical professionals’ work with respect to clinical evaluation, audiological testing and questionnaire administration. The results obtained by the automated processes can be easily evaluated by a specialist and stored digitally. This automation helps save time, greatly simplifies the overall evaluation of a patient, and avoids the necessity of collecting paperwork that is difficult to manage. Besides, the data produced by the app regarding the health status of a patient, is stored locally in the smartphone, which can be easily protected using native security measures, such as smartphone encryption (e.g., [11]);
– The application has a functionality which enables a physician to monitor and trace their patients’ condition remotely, whenever the patient shares his/her analyses with the physician, i.e., the physician is able to analyse if there are any significant variations with respect to the previously stored examinations and can decide if it is necessary for the patient to return for a specialist check. It should be remarked that the device has very advanced audiometric capabilities and the app provides detailed automated reporting which describes the severity of hearing loss and classifies it into three types (sensorineural, transmissive and mixed). This may also enable a patient to control their hearing autonomously during some periods and may help them decide when to contact a specialist (when the app indicates a worsening in her/his condition);
– The possibility for a patient to monitor his/her condition could be fundamental during a crisis, instead of waiting for an appointment with the doctor to perform all the required analyses;
– The app can be used for the collection of data for new studies regarding tinnitus, given that the data obtained from the questionnaires and the tests can be easily shared with doctors. In such a way, multiple groups of patients can be included in a study; by creating a database with a large number of patients, the results of patients from different regions could be easily compared, without the necessity of direct contact between the patient and the doctor.

The paper is organised as follows. Section 2 introduces background information on tinnitus, its clinical evaluation, the audiological and psychoacoustic examinations, and the questionnaires for measuring the impact of tinnitus on different aspects of the patients’ quality of life. Section 3 summarises the related work on apps and devices specifically dealing with tinnitus. Section 4 describes the ad-hoc developed hardware device, in terms of its hardware and firmware, plus ergonomics considerations. Section 5 discusses the first version of the app, how the automated examinations were implemented, and two experiments concerning the quality of the automated reporting procedure and the user experience with the app. Based on the results of the experiments, Section 6 presents an improved version of the app with the improved user experience. Finally, Section 7 ends the paper with conclusions regarding the app, the limitations and a discussion on future lines of research.

## 2 Background

In this section, a brief review of the problem of tinnitus is initially presented, from its definition to the difficulties still encountered today. This is followed by a description of the most common current process for clinically evaluating tinnitus, with emphasis on audiometry and acufenometry.

### 2.1 Tinnitus

The scientific literature offers several definitions of tinnitus. According to Cusimano et Martines [5], “[…] tinnitus are sound sensations perceived by the individual, not supported by external sources, acoustic or electric, nor acoustic apparatus, that are caused by his/her activities or by alterations of the mechanisms of sensory processing […]”. Recent statistics provide several data on the epidemiology of tinnitus, ranging from 5–25% (according to American authors) to 9–29% (according to European authors) to 7–34% (according to West Pacific authors), with a minimum of 5% and a maximum of 42% [6]. Furthermore, its prevalence increases with age and is more prominent in males than females [6].

Tinnitus can impact on the overall health and social well-being of patients [7]. People with tinnitus often suffer from distress, depression, sleep disturbance, anxiety, poor concentration, irritability or frustration. The American Tinnitus Association (ATA) conducted a survey for its members in 2014, to evaluate how ATA members experience tinnitus [8]. Over 1100 people responded to the survey: the majority (about 36%) barely noticed it or found it slightly annoying (without a significant impact on the quality of life); others reported sleep problems, trouble concentrating and anxiety (respectively, 18%, 16% and 13%); the remaining – with smaller percentages – reported social isolation, ongoing depression and inability to work.

The classification of tinnitus is still an open question [12]. Out of all types, clinical classification is the simplest: it distinguishes between subjective and objective tinnitus. Subjective tinnitus (also known as “intra-auditory”) has a neurophysiological origin and is very common. Objective tinnitus (also known as “extra-auditory”) can be generated from vascular, muscular or respiratory sources and also from the temporomandibular joint. On the basis of the most recent acquisitions, in this article we focus on a more detailed “pathogenic” classification of subjective tinnitus [13]:

**Conductive** in the case of myocardial twitch of the middle ear or disturbance of tubal ventilation;

**Sensorineural** when we have an anomaly in the inner ear or sensory organ (cochlea and associated structures) or vestibulocochlear nerve (cranial nerve VIII) or neural part.

**Central** when in presence of primary intracranial tumor, multiple sclerosis, traumatic brain injury and closed secondary “ghost sound”.

### 2.2 Clinical evaluation

The diagnostic procedure normally consists of the following three phases:

1. Diagnosis and accurate audiological examinations (Audiometry, see Section 2.2.1, and others like Tympanometry and Otoscopy that are not included in our approach);
2. Psychoacoustic measurement of tinnitus (Acufenometry, see Section 2.2.2);
3. Evaluation of disability (Questionnaires, see Section 2.2.3). Together with the previous two analyses, this is another essential phase, since it allows to identify patients that are seriously incapacitated by tinnitus [14, 15].

#### 2.2.1 Audiometry

Commonly, audiometry tests are based on a key hearing test called Pure Tone Audiometry (PTA), where a pure tone is a sound having a single specific frequency [13]. The intensity of the pure tone is considered to be the loudness of the sound which is measured in decibels (dB). The most common dB scales in audiometry are: the dB HL (hearing level) scale, the dB SPL (sound pressure level) scale, and the dB SL (sensation level) scale. By far, the most common one used in audiology is the dB HL scale. Accordingly, a PTA is the procedure that uses pure tones to assess an individual’s hearing. Pure tones are generated at different frequencies and intensities by an audiometer and presented to the patient via headphones. Depending on the transducer, the audiometry can be either air-conducted or bone-conducted, as detailed below.

##### 2.2.1.1 Air conduction audiometry

An air conducted signal is defined as a sound wave travelling through air; this is the means by which humans typically hear most sounds. This mode of signal presentation assesses the entire auditory system. Thus, when hearing loss is detected during air conduction, further tests become necessary to determine which part(s) of the auditory system are dysfunctional [13].

The general procedure for air conduction pure tone audiometry goes as follows. The patient is instructed to listen carefully for a beeping sound (pure tone): when heard, even if very softly, he/she is asked to give a positive response and press the answer button or raise his/her hand. Pure tones are then presented to the patient, initially at an intensity level that it is assumed can be heard quite well. After the patient demonstrates a good understanding of the task, the intensity (loudness) of the tone is decreased in 10–15 dB steps, until the patient no longer responds. The intensity is then raised in 5 dB steps until the patient responds, decreased again and increased again in 5 dB steps until the patient responds for the last time. The lowest audible intensity is then defined as the patient’s threshold for the particular frequency. This method is described as the “modified Hughson–Westlake ascending-descending paradigm” [16]. This routine is repeated for all test frequencies in one ear, then again in the other ear. Usually, the test frequencies are 125 Hz, 250 Hz, 500 Hz, 1 KHz, 2 KHz, 3 KHz, 4 KHz, 6 KHz and 8 KHz. Such a procedure then establishes an air conduction pure tone threshold curve for each ear called audiogram.

In the presence of hearing loss, bone conduction audiometry must be performed [17].

##### 2.2.1.2 Bone conduction audiometry

Bone conduction pure tone testing stimulates the cochlea directly, bypassing the outer and middle ear. This type of testing is used to determine whether a hearing loss is due to a cochlear/neural deficit or an outer or middle ear dysfunction.

Bone conduction pure tone audiometry is performed using the same modified Hughson–Westlake method as in air conduction audiometry, but the tones are presented via a bone conduction headset. This is comprised of a bone oscillator affixed to a metal band which can be worn over the head in a similar fashion as standard headphones. The bone oscillator is typically placed on the mastoid process [17].

If bone conduction pure tone thresholds agree with air conduction thresholds, the loss is determined to be related to the cochlea or higher neural processes and is termed “sensorineural” [13]. If, on the other hand, bone conduction thresholds are better than air conduction thresholds, a “conductive” hearing loss is present: the cochlea, when stimulated directly, responds at lower intensities (i.e.,“better”) than when it is stimulated via the outer and middle ear, meaning something in the outer or middle ear is reducing the sound intensity that reaches the cochlea. If there is both a sensorineural and a conductive component, the loss is termed as a “mixed” hearing loss.

#### 2.2.2 Acufenometry

Acufenometry is used to determine the frequency and intensity of tinnitus. Several methods can be adopted, e.g., pitch matching, method of adjustment, forced-choice double-staircase adaptive procedure [18, 19].

The standard measure for tonal tinnitus is pitch matching, easily understandable by patients and technically straightforward to implement in the app. In this method, patients are asked to compare the frequency of a test-sound (i.e., a pure tone) with that of tinnitus. Two tones are presented alternately to both ears so that each is heard 4–5 times; the frequency is changed (increased or decreased) until the patient finds the one that is the closest to that of tinnitus. Intensity is therefore established by comparing the test-sound with that of tinnitus. A pure tone at the previously identified frequency is first sent at subliminal levels to the other side ear. Then, the intensity is increased by 5 dB until the patient hears it. In this way the “threshold of perception” of a signal is established and taken as the reference level of 0 dB. By increasing the intensity by 5 dB steps, the patient is asked to report when the sound level completely masks that of tinnitus. The frequency and intensity – reported in this way by the patient – represent the result of the acufenometry.

#### 2.2.3 Questionnaires

As mentioned earlier, people with tinnitus often experience hyperacusis, insomnia, distress, depression, sleep disturbance, anxiety, poor concentration, irritability or frustration [20–22]. For these reasons, the clinical evaluation, together with the audiological tests, must be supported by the use of specific questionnaires that are usually self-administered. Those that are currently provided by the app are described below.

##### 2.2.3.1 Pittsburgh sleep quality index (PSQI)

The Pittsburgh Sleep Quality Index (PSQI) is a self-administered questionnaire which assesses sleep quality and disturbances over a 1-month period [23]. It is made up of nineteen individual items that measure seven domains: subjective sleep quality, sleep latency, sleep duration, habitual sleep efficiency, sleep disturbances, use of sleep medication, and daytime dysfunction over the last month. A (somewhat) complex scoring procedure yields to the following conclusions: if the total score is ≤5, a good sleep quality is detected; if the total score is >5, it indicates poor sleep quality.

##### 2.2.3.2 Khalfa hyperacusis questionnaire

The Khalfa Hyperacusis Questionnaire is a tested and validated tool, suitable to quantify and evaluate various hyperacusis symptoms. It is made up of 14 questions, each with four possible answers, namely: “no”, “rarely”, “often” and “always”. The scoring procedure yields to a total score: if greater than 28, the patient experiences severe hyperacusis; if greater than 16, it is a mild hyperacusis; in the remaining case, the result is normal [24].

##### 2.2.3.3 Tinnitus handicap inventory (THI)

The Tinnitus Handicap Inventory (THI) [25] is a self-administered questionnaire which evaluates the impact of tinnitus on the quality of life. It is made up of 25 questions, each with 3 possible answers, namely: “no”, “sometimes” and “yes”. According to the score, the following grades of disorder can be identified [26]:

– Grade 1 – very slight tinnitus – (THI score 0–16): Tinnitus is perceived only in a quiet environment, masked very easily. It does not disturb your sleep or daily activities;
– Grade 2 – mild tinnitus – (THI 18–36): Easily masked by noise environment and forgotten during the activities. Occasionally it may interfere with sleep but not with common tasks;
– Grade 3 – moderate tinnitus – (THI 38–56): It can also be perceived in presence of background noise or ambient noise although the daily activities can be carried out regularly. It is less relevant when you are focused. Sometimes interferes with sleep and with the activities carried out in conditions of silence;
– Grade 4 – severe tinnitus – (THI 58–76): The tinnitus is perceived almost always, is masked rarely or never, disrupts sleep and may interfere with the ability to carry out normal daily activities. Tinnitus also hurts with daily activities in conditions of silence;
– Grade 5 – catastrophic tinnitus – (THI 78–100): All the symptoms of tinnitus are worse than the previous level and can be associated with psychiatric illness.

## 3 Related work

As previously mentioned, tinnitus is a very common problem, the treatment of which is not very well defined as there are still no medically approved devices for it. This has aroused the interest of the scientific community, but there are still not many recent research articles in the literature addressing this problem [27, 28].

With respect to the design and development of electronic devices, one of the few recent studies that attempt to address the problem of creating a hardware device oriented towards the treatment of tinnitus can be found in the master’s thesis of Berbon Pedrina, A. [29], whose objective was to design “a portable multi-modal stimulator that is able to provide bilateral acoustic and electrical stimulation simultaneously”. However, as far as the device is concerned, only a brief description without images of the hardware device is included, and it is emphasized that it is a prototype based on the Arduino Uno board, so that its use in health care environments and its industrialization is still far away. Other recent and noteworthy articles discussing the hardware device do not focus on the definition of a new device, but rather on how the use of one or more existing devices can help reduce the symptoms of the disease, as in the paper presented in [27].

As a general rule, whenever there is a hardware device, there must be software that allows for the control and monitoring of everything that the device is capable of. Until a few years ago, in most medical equipment, this software was embedded in the device itself and could be purchased as a whole. However, the use of external devices, such as ordinary computers or smartphones to run such software is increasing considerably in recent years. This is due to advances in computing, which make it possible to process the signals captured by the hardware device at a very affordable price, since virtually any device today can do so and a significant part of the hardware device components are eliminated, making the final product considerably cheaper. For this reason, the use of smartphone apps in the medical context is increasing [30].

In most cases, the apps are developed for a specific disease or purpose; in some cases these apps are medically assessed and in few cases there are actual reports about their development.

In the paper presented in [31], a complete comparison of more than 30 existing apps (available for iOS and Android) is shown, although not all of them are oriented towards the evaluation or treatment of tinnitus, but most of them are oriented towards hearing tests.

Thus, numerous apps that perform hearing tests are available [32], even if significant research in this field – particularly in terms of assessment – is still needed [33]. Nevertheless, with respect to tinnitus, a small set of apps has been developed so far, e.g., [34] presents a smartphone app whose purpose is to promote skills that help manage and cope with tinnitus; the app described in [35] enables researchers to collect longitudinal data under real-life conditions and with high cost efficiency (e.g., in [36] it is used to monitor the circadian variations of tinnitus). Finally, one of the most recent applications is TinnituSense, presented in [37], an app for EEG recordings and visualization which can be very relevant for tinnitus research, but it is currently unavailable, so its evaluation could not be performed.

The conclusion drawn from the related research is that there is a need to investigate and work on the creation of a device that can be medically approved for the treatment of tinnitus. It is intended to make a control and monitoring software based on a mobile app with two main objectives: (i) to reduce manufacturing costs without losing the data processing capacity, (ii) to facilitate the control of end users, since the vast majority of users are used to interact with mobile apps.

## 4 The device

This section presents the aspects related to the device that has been designed and built specifically for the realization of this work, initially detailing the technical aspects and, subsequently, presenting details related to the considerations that have been taken into account to optimize its ergonomics.

### 4.1 Electronics design and manufacturing

Under the framework of the project, a dedicated electronic device has been designed and three prototypes were developed, each having an increased reliability and levels of integration. The initial prototype is discussed in [38, 39]. Hereafter, the most recent one is described. The device has the capability to generate a pure tone at any frequency and intensity (loudness), and can be managed through a smartphone. In addition to the inherent functionalities for generating pure tones, this device has been designed to boost its capabilities and future usage, through the following main characteristics. First, the absence of a battery or external power supply: it only needs to be physically connected to a smartphone. Such a feature greatly reduces costs and maximises its ease of use. Second, it is smartphone and operating system independent, since it is controlled through a simple and generic interface based on serial port. Third, affordable electronic components have been selected for its construction, without diminishing the quality of the final product, making the device accessible to both medical staff and patients.

**Figure 1:**
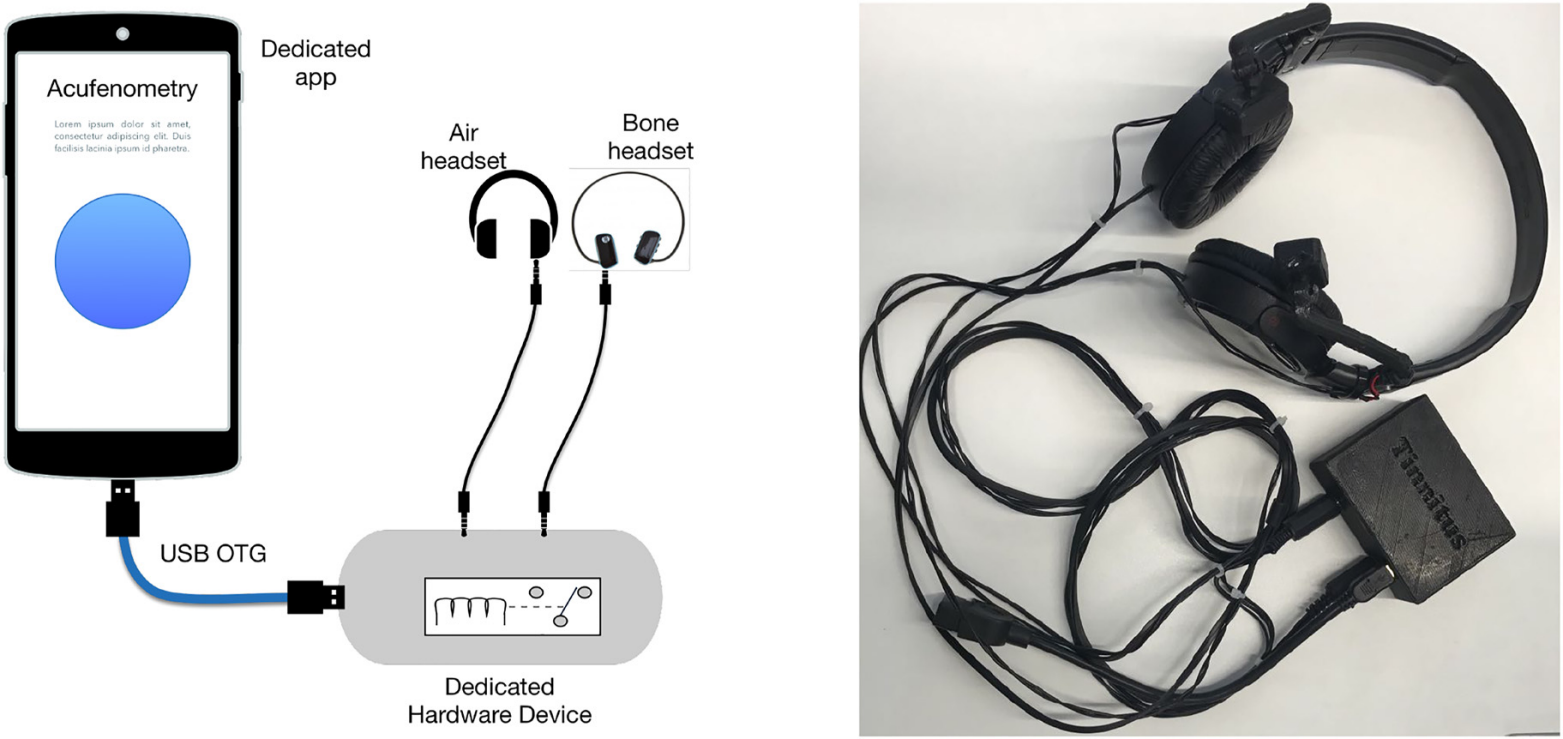
The dedicated device acts as a external peripheral controlled by the app.

Figure 1 shows the interconnection scheme between the smartphone, the device and the external audio outputs (air/bone). In this schema, the device acts as an external peripheral, controlled by the smartphone and its dedicated app, connected through a USB On-The-Go (OTG) port that serves for both controlling (set up and execute commands) and supplying power to the device. Furthermore, the external outputs of the hardware device are connected to air headset and bone conduction transducers through a standard audio cable and a 3.5″ jack. In the project, a prototype of headphone that integrates both air and bone conductors has also been developed (see Figure 1).

**Figure 2:**
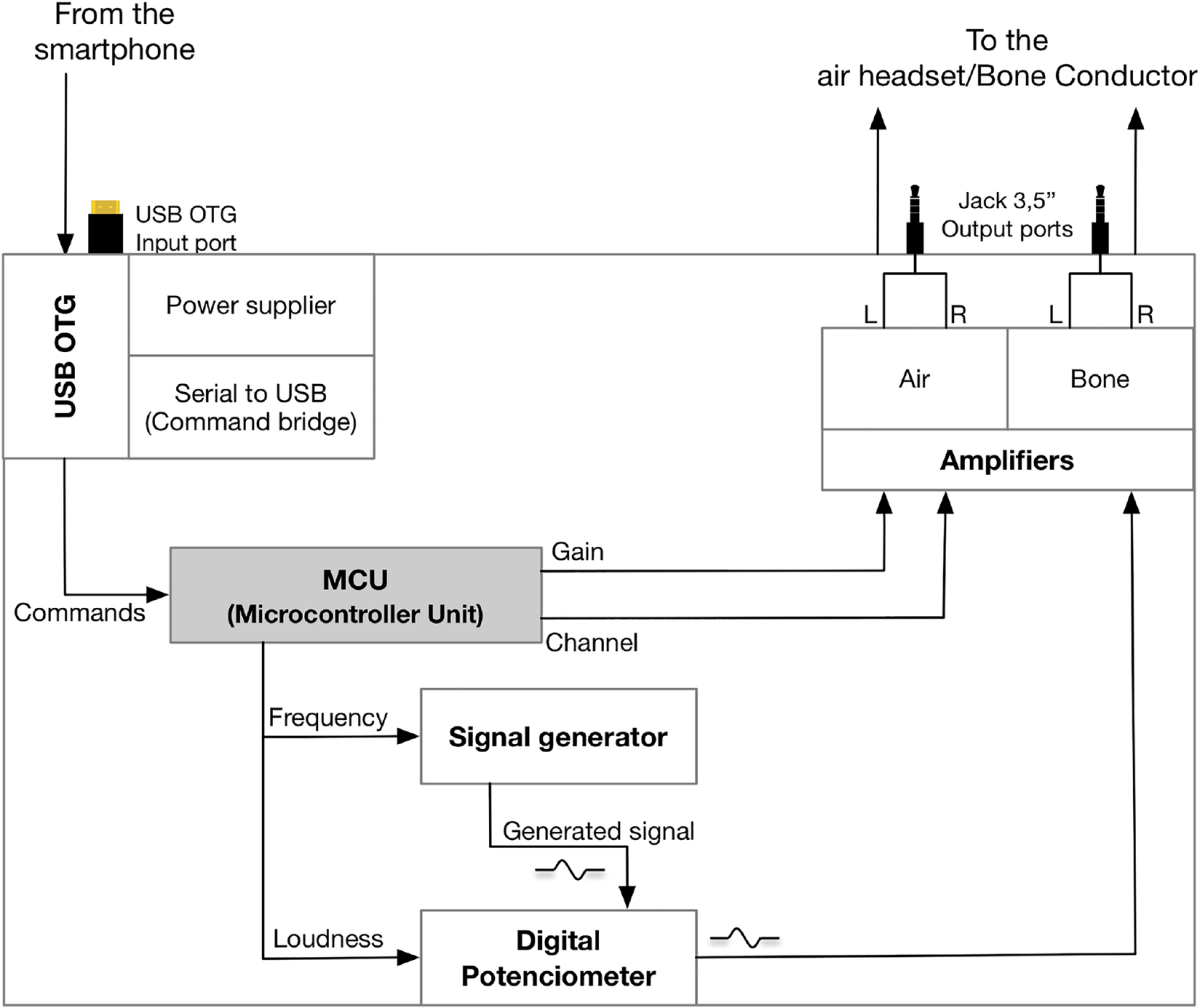
Components diagram of the device, interconnections and control flow.

Figure 2 shows the general schema of the device. The device includes three ports, one input port for receiving information and a power supply from the smartphone and two output ports for its connection to the headsets (air and bone). The connections are standard (USB and 3.5″ jacks). As for the input port, it has a USB OTG with a twofold functionality. Firstly, it provides the power supply required for the device to operate correctly – with a consumption between 1.1 W (maximum) and 0.1 W (standby) – greatly simplifying its maintenance and usage. Secondly, the USB OTG port also acts as a serial port (serial over USB) to enable the smartphone to control the device. Receiving commands directly from the smartphone allows it to be used with any phone model and operating system. Likewise, the system can be used with almost any kind of headset (air/bone), i.e., able to reproduce frequencies ranging from 125 Hz to 8 kHz.

The control of all components is performed by the firmware stored and executed in the microcontroller Unit (MCU). The firmware receives commands coming from the smartphone through the serial port over USB, as shown in Table 1. Once a command is received from the smartphone, it is analysed by the MCU, which executes the following tasks. First, the MCU selects the output to be activated (either air or bone) through a separated amplifier. Then, it sets up the required levels of intensity through a digital potentiometer. Then, the MCU sends the frequency of the signal to be generated to the signal generator. The signal passes through the digital potentiometer, to the amplifiers, up to the air or bone conductor.

**Table 1:** List of commands that the smartphone can send to the device.

ID	Command	Arguments
0	Air-left channel	
1	Air-right channel	
2	Bone-left channel	Frequency: 125–8000
3	Bone-right channel	Potentiometer: 0–256
4	Air-left-right channel	Gain: 0–3
5	Bone-left-right channel	
6	Air-bone-left-right channel	

As for the calibration of the transducers, three fundamental aspects were taken into account. First, the same transducer models were used. Second, the behaviour of the transducers is the same regardless of the mobile device to which they are connected. Third, there is a limited range of values in which the transducer intensity may vary. Accordingly, when initially calibrating the chosen transducer model, the power needed to equate the bone and airway transducers was measured, for each of the possible intensity values. Furthermore, the app implements a function that, given the required intensity, frequency and ear, returns the values to be set for the digital potentiometer and gain. Such a function is clearly tailored to the specific air and bone transducers used in the hardware, however, the device must be simple to use and affordable for patients, which meant that a compromise had to be made between precision and costs. Accordingly, the total cost of all components and transducers does not exceed 80€. However, in mass production, its cost could be even lower than 30€.

### 4.2 Ergonomics considerations

For use at home, the device must be easy to place and wear, without the assistance of an audiologist. Four users have been involved in the following study:

– we asked the user to wear the headset and check for any difficulties or errors;
– a sound was generated using the bone headset, for the right ear;
– the users were asked if he/she could hear the sound and if it came from the right ear. The users with long hair were asked if long hair interfered in any way with the use of the device;
– it was observed whether the positioning of the bone transducer was correct.

The results show that most of the users had issues in identifying the correct way to wear the headset. Furthermore, the positioning of the bone headset was correct, the sound was audible. One of the users found that the cables caused discomfort while long hair did not interfere with the device.

Hence, the following improvements were devised:

– the integrated headset should:
  – the correct position of the headset should be clearly indicated (e.g., by printing the “L” and “R” letters on the corresponding side of the headset);
  – all the cables must be integrated into a single cable or bluetooth must be used instead;
– the app should give a brief set of instructions concerning how to wear the headset, before starting the audiometry and acufenometry.

## 5 First iteration

The objective of this section is to present the first version of the application developed and the results obtained with this version. Therefore, in the first subsection, all the aspects related to the mobile app are detailed, including automated audiometry, acufenometry and user questionnaires. Subsequently, we present the evaluation carried out in this first iteration of the work.

### 5.1 The app

The functionalities of the app, needed to conduct a thorough evaluation of tinnitus, are described in Subsection 2.2. The app and the device are described in Section 4 and used for automated audiometry in (Subsection 5.1.1) and for acufenometry in (Subsection 5.1.2). Furthermore, the application automatically assesses the questionnaires regarding sleep quality, hyperacusis and impact of tinnitus on the quality of life (Subsection 5.1.3).

#### 5.1.1 Automated audiometry with reporting

The app implements the Hughson–Westlake process described in Section 2.2.1, for both air and bone conduction audiometry, with the following exceptions caused by the fact that the app is used autonomously: (i) the patient touches a button placed in the centre of the smartphone to signal that he/she has heard the sound; (ii) the decrement/increment of intensity is not performed. The process returns a matrix of intensities (audiogram), i.e., when the patient heard the tone at specific loudness (hereafter called *PTA*), for both ears and both ways (i.e., air and bone), for all investigated frequencies. The matrix of intensities is then given as an input for the automated audiometric reporting procedure.

**Figure 3:**
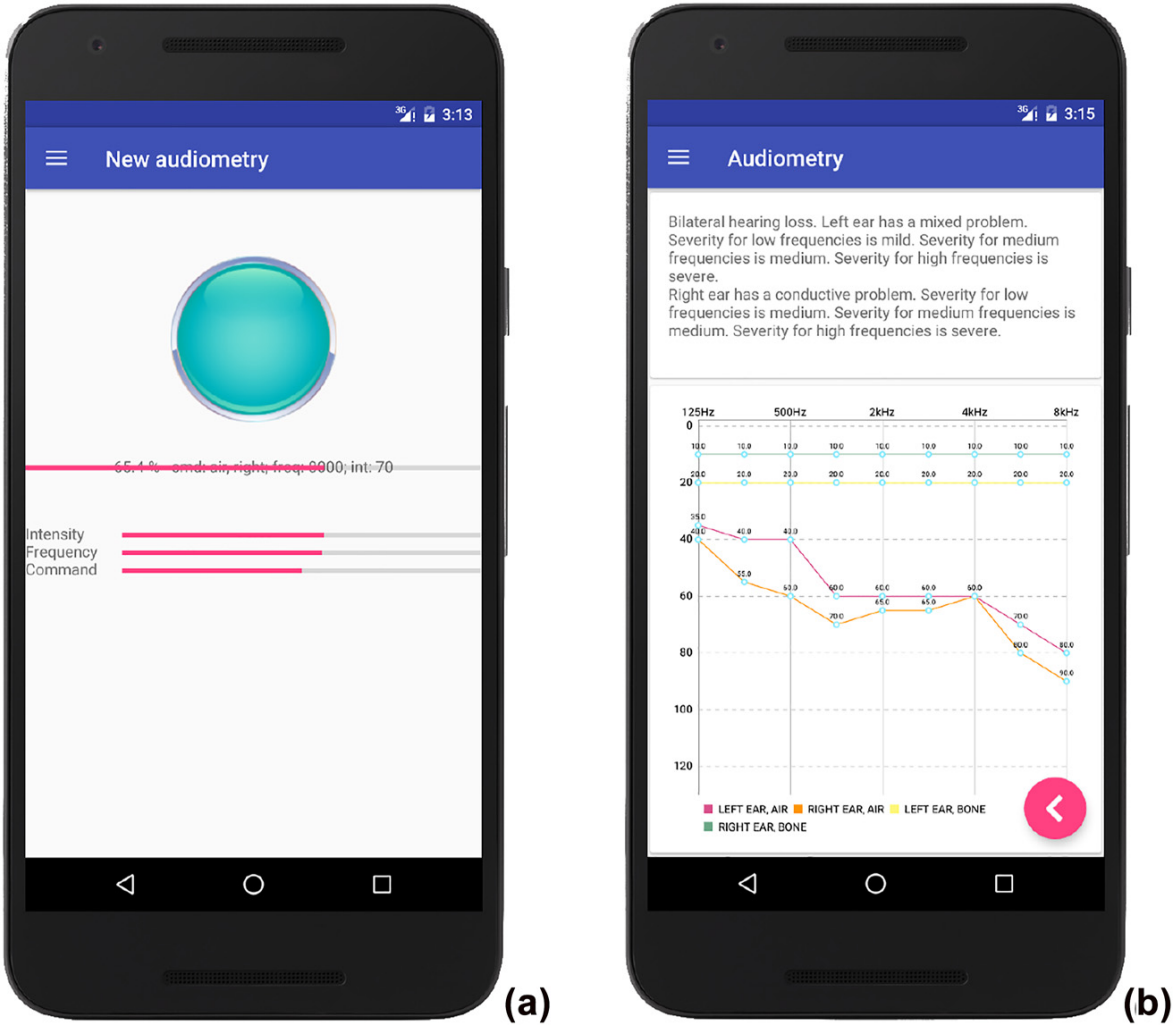
Air/bone conduction audiometry in the developed app. (a) An audiometry in progress where different pure tones are sent through the air/bone conductor at different frequencies and levels of loudness, and (b) the result is provided in the form of an audiogram with automated reporting.

Figure 3 shows the interfaces for executing audiometry. Figure 3(a) indicates the central button that the patient has to press to signal that he/she heard the sound; on the bottom the progress of the audiometry can be viewed and Figure 3(b) is the interface for displaying results (automated reports are shown on the top; the audiogram is on the bottom).

The automated audiometric reporting procedure works as described in Algorithm 1: initially, it cycles on both ears and all frequencies, it recalls the intensities for both air *I*_*a*_ and bone *I*_*b*_ conduction (lines 4–5); then deduces the type of problem and its severity (lines 7–9); all information on frequency ranges is collected, i.e., low frequencies ∈ {125 Hz, 250 Hz, 500 Hz}, medium frequencies ∈ {1 KHz, 2 KHz}, and high frequencies ∈ {3 KHz, 4KHz, 6KHz, 8 KHz} (line 12); finally, the source of the problem is identified (line 14).

**Algorithm 1:** Automated audiometric reporting.

1: **procedure** AAR(PTA)
2: **for all** ear ∈ {LEFT, RIGHT} **do**
3: **for all** freq ∈ {125 Hz, …, 8 KHz} **do**
4: *I*_*a*_ = PTA[ear, freq, AIR]
5: *I*_*b*_ = PTA[ear, freq, BONE]
6:
7: type[freq] = ProblemType(*I*_*a*_, *I*_*b*_)
8: **if** (type[freq] == NORMAL) **then**
9: severities[freq] = NO LOSS
10: **else**
11: severities[freq] = Severity(*I*_*a*_)
12: **end if**
13: **end for**
14: ⟨ problem[ear], severity[ear] ⟩ = SumUp(type, severities)
15: **end for**
16: ⟨where, problem, severity⟩ = Location(problem, severity)
17: **return** ⟨where, problem, severity⟩
18: **end procedure**

The decision concerning the type of problem – summarised in the procedure called ProblemType – is quite straightforward and therefore not reported in detail. It is implemented as described in Subsection 2.2.1: if both intensities are below a threshold, i.e., 25 dB, there is no hearing loss. If bone conduction is worse than air conduction, there is an error in the audiometry; if bone conduction agrees with air conduction (i.e., the difference is ˂20 dB) then the problem is considered to be sensorineural; if it is on the contrary, i.e., bone conduction is better than air conduction, the problem is considered to be conductive.

The severity of hearing loss is instead deduced by a procedure called Severity, that simply implements the thresholds defined in [40]: (i) no hearing loss, *PTA* ∈ [0 d*B*, 25 d*B*); (ii) mild, *PTA* ∈ [25 d*B*, 40 d*B*); (iii) moderate, *PTA* ∈ [40 d*B*, 70 d*B*); (iv) severe, *PTA* ∈ [70 d*B*, 90 d*B*); (v) deaf, *PTA* ∈ [90 d*B*, +∞).

Once the type and severity of the problem are defined for all frequencies, a summative interpretation of the phenomenon (for each ear) is provided by the procedure called SumUp. Firstly, if all measurements are normal, the procedure concludes that the audiometry (for that ear) is normal. Otherwise, if all types are sensorineural, then the ear has a sensorineural problem; if all types are conductive, then the ear has a conductive problem; otherwise, the problem is mixed. Then, the severities are qualified into the low, medium and high frequency ranges. Hence, in summary, the procedure returns the problem (either NORMAL, SENSORINEURAL, CONDUCTIVE or MIXED) and the severity (either NO LOSS, MILD, MODERATE, SEVERE and DEAF), for the three ranges.

Finally, the algorithm calls the Location procedure: if the SumUp procedure detects problems in both ears, the overall problem is stated to be BILATERAL, otherwise, the problem is either in the LEFT or the RIGHT ear.

#### 5.1.2 Automated acufenometry

**Figure 4:**
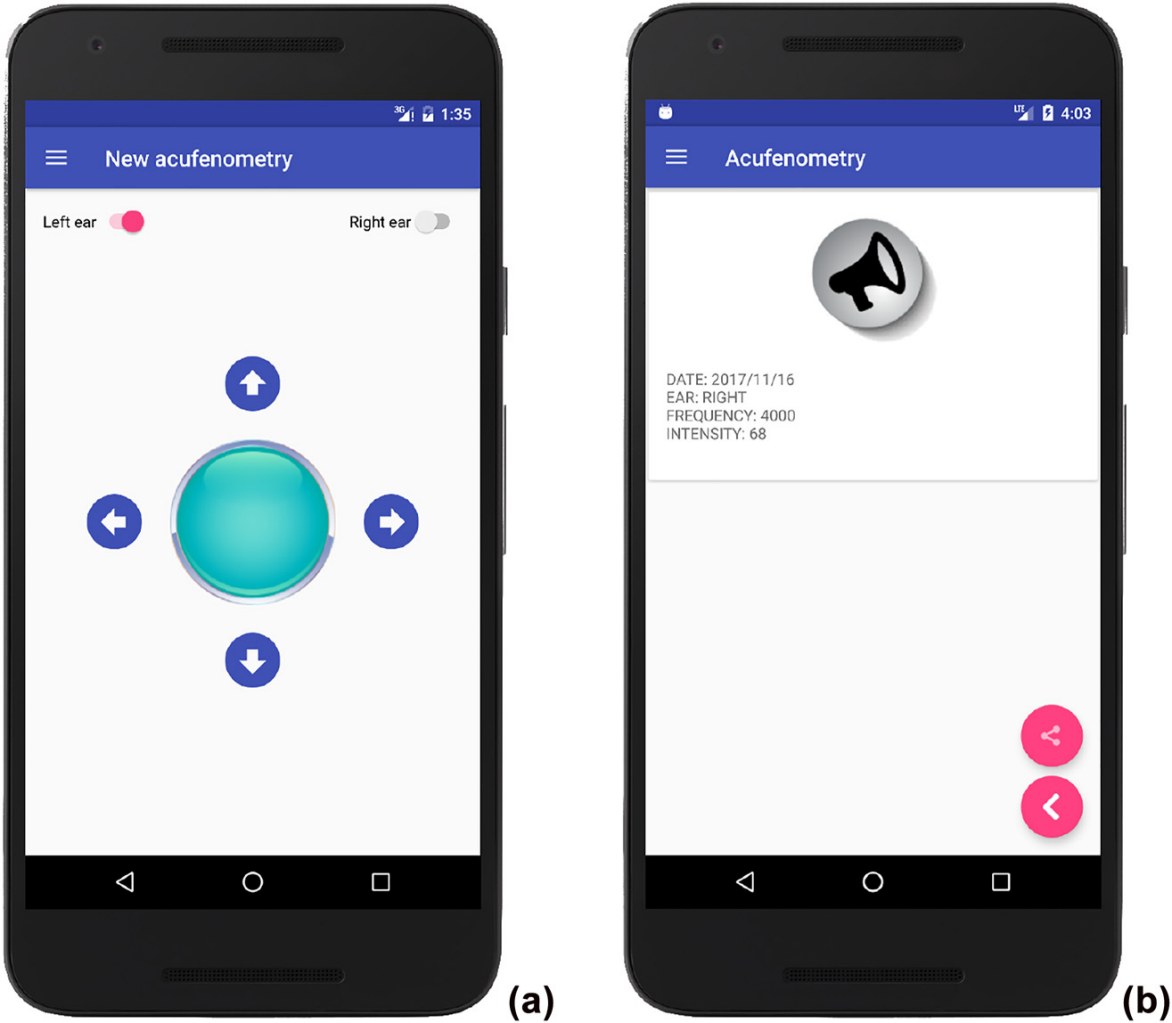
The acufenometry test implemented in the app: (a) the interface for pitch and loudness matching and (b) the returned feedback.

The app implements the acufenometry procedure described in Subsection 2.2.2, without detecting the level of the “threshold of perception”. Figure 4(a) shows the interface used for performing acufenometry: the switches placed on the top can be used to select which ear experiences tinnitus, the horizontal/vertical arrows change the frequency/intensity of tinnitus, while the central button can be tapped to confirm that the emitted sound actually resembles tinnitus. Figure 4(b) is the feedback returned by the app.

#### 5.1.3 Questionnaires

**Figure 5:**
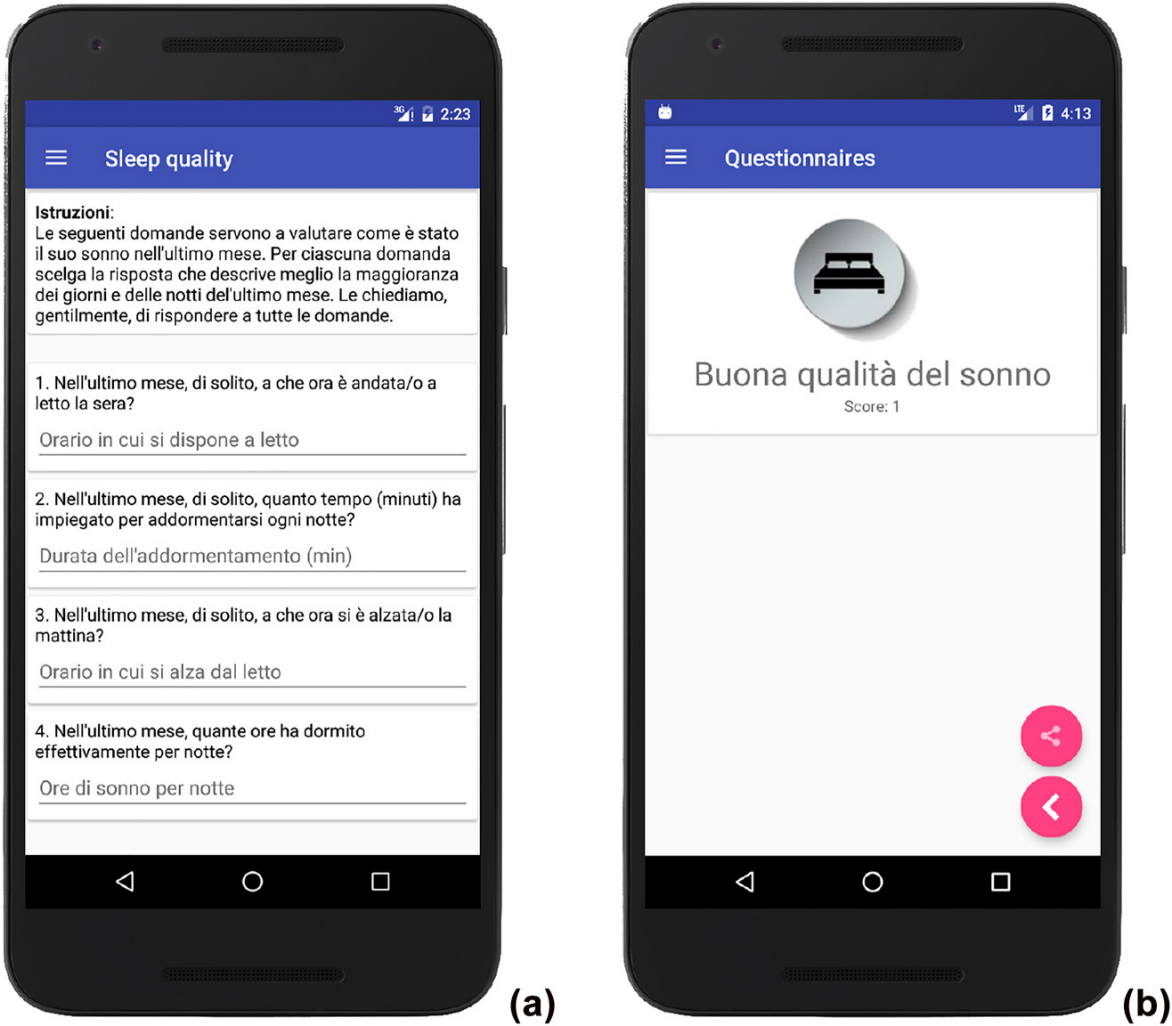
Questionnaires: (a) PSQI administration and (b) app feedback (in Italian).

The PSQI, Khalfa and THI questionnaires are implemented and automatically scored by the app. A simple ad-hoc XML document defines and enables the app to display the questionnaires, whereas automated scoring is performed by an ad-hoc class. This choice of design makes it possible to include different languages and easily add new questionnaires that may be used for further examination, e.g., depression or other aspects related to tinnitus. Figure 5(a) and (b) show – as a matter of example – the PSQI questionnaire and the related scoring (in Italian language), as proposed by the app. Similar interfaces are available for the Khalfa and THI questionnaires.

### 5.2 Evaluation

This subsection describes the evaluation performed as part of the first iteration, including a report on the usability of the system.

#### 5.2.1 Automated audiometric reporting

The quality of the automated audiometric reporting procedure was evaluated as follows: the data regarding several clinical audiometries were manually entered into the app (i.e., without using the device), then the returned reports were compared with the corresponding reports written by physicians.

In particular, we selected the audiometries performed on patients with tinnitus in the “Otorinolaringoiatria” ward of the Hospital of L’Aquila (Italy), during the period October, 2013 – July, 2016. The archive consisted of a total of 89 audiometries: 3 conductive, 11 mixed, 55 sensorineural, the remaining 20 did not exhibit any hearing loss. Given that conductive and mixed audiometries were not very frequent, the sample used for comparison was made up entirely of conductive and mixed audiometries, plus a 20% random sample of the sensorineural ones (i.e., other 11 audiometries). Accordingly, a total of 25 audiometries were used for comparison. For each of the 25 selected audiometries, the same audiogram was replicated on the app so that the app could automatically generate the audiometric report. The results show that all the automated audiometry reports were correct, a few of them were even more detailed than those written by human experts.

One possible improvement was identified in the automated reporting: an audiometry showing a problem only for one frequency is usually reported as an “acoustic hole”. Since this term is not provided by the app, it might be added in the next release.

#### 5.2.2 Usability

The reason for choosing to evaluate one type of functionality over another is related to the stage of development of the project; what can be evaluated, which experts are available, as well as the time constraints and the project’s available resources [41]. For the first version of the project, in accordance with the state of the art, we decided (i) to ask two usability experts for a heuristic evaluation in order to generate the initial number of potential usability problems (Section 5.2.2) and then (ii) to enquire users about the actual problems (Section 5.2.2).

##### 5.2.2.1 Expert-based review

The most popular type of expert evaluation is heuristic evaluation [41]. Two evaluators – senior researchers in the Human-Computer Interaction research field – were involved in the expert-based review: they used a specifically designed check-list to evaluate mobile interfaces, which reuses around 70% of literature heuristics, the rest derives from best-practices and recommendations for mobile interfaces [42]. Accordingly, Table 2 summarises the results: the table lists the evaluated items and the corresponding rating, i.e., a numeric value (Likert scale) with the following meanings: 0 = *poor*, 1 = *acceptable*, 2 = *good*. The *NA* rating was used when the heuristic did not apply to the function of the app (e.g., shopping).

**Table 2:** Expert-based evaluation result.

Item	E1	E2
Visibility of system status		
System status feedback	2	2
Location information	NA	
Response times	2	1
Selection/input of data	1	1
Presentation adaptation	NA	
Match between system and the real world		
Metaphors/mental models	1	1
Navigational structure	2	2
Menus	2	2
Simplicity	2	2
Output of numeric information	NA	
User control and freedom		
Explorable interfaces	0	1
Some level of personalization	NA	
Process confirmation	1	1
Undo/cancellation	0	1
Menus control	2	2
Consistency and standards		
Orientation	1	1
Design consistency	2	2
Menus	2	2
Input fields	2	2
Naming convention consistency	2	2
Menu/task consistency	2	2
Functional goals consistency	1	1
System response consistency	2	2
Error prevention		
Error prevention	1	1
Fat-finger syndrome	1	1
Recognition rather than recall		
Memory load reduction	2	2
General visual cues	1	2
Input/output data	2	1
Menus	2	2
Navigation	2	2
Flexibility and efficiency of use		
Search	1	0
Navigation	2	2
Aesthetic and minimalist design		
Aesthetic and minimalist design	1	2
Multimedia content	1	1
Icons	2	2
Menus	2	2
Orientation	2	1
Navigation	2	2

**Table 2:** (continued)

Item	E1	E2
Help and documentation		
Help and documentation	1	1
Help users recognize, diagnose and recover from errors	0	0
Skills		
Skills	0	0
Pleasurable and respectful interaction		
Pleasurable and respectful interaction	1	1
Input data	1	1
Shopping	NA	
Banking and transaction	NA	
Privacy		
Privacy	NA	

The agreement between the two evaluators was measured through a Cohen’s Kappa [43], in order to assess the consistency of the evaluations. The result is *K* = 0.639, i.e., a good agreement between the two experts while evaluating the app. The median rating is 2, the average rating is 1.43, thus showing that the app should offer average/good usability.

Few suggestions about how to improve usability were given by the experts: (i) clearly show the goals of each functionality (i.e., audiometry, acufenometry and questionnaires); (ii) add a search facility, even if it may not be necessary given the shallow navigational structure. The first point was immediately implemented in the app: now a help dialogue opens every time a user selects an audiometry, an acufenometry or a questionnaire, thus explaining the goal of the functionality.

Given the encouraging results of the expert-based evaluation, a user-based evaluation has been conducted, as reported below.

##### 5.2.2.2 User experience case study

For this study, quantitative data were gathered for the following metrics: (i) System Usability Scale (SUS) [44], (ii) Single Ease Question (SEQ) [45] and (iii) Expectation Measure (EM) [46].

First, SUS, a reliable tool for measuring usability, was employed. It consists of 10 items with five response options per item. The tool rates a system with a score ranging from 0 to 100, the higher the score the more useable the system is. It can be used to evaluate a wide variety of products and services, including mobile apps.

The SEQ is a 5-point rating scale that is used to assess the degree of ease or difficulty that a user experienced in accomplishing a particular task. The higher the value on the rating scale, the easier the task is. Users were asked to rate the following three tasks: (T1) performing an audiometry, (T2) performing an acufenometry, and (T3) completing a questionnaire. On the other hand, EM results were compared with the results of the SEQ, to check the difference between the users’ expectations regarding the level of difficulty and the real experience. EM has the same 5-point rating scale. Thus, before the users actually did any of the tasks, they were asked to rate how easy/difficult they expected each of the tasks to be, simply on the basis of their understanding of the tasks. For instance, for task T1, the questions were:

*In* a scale from 1 to 5, where 1 = very difficult, 2 = difficult, 3 = normal, 4 = easy, 5 = very easy, **[EM]** how easy do you think the audiometry process will be? **[SEQ]** how easy did you find the audiometry process?

A total of 26 users participated: their average age was 39 with a standard deviation of 15, ranging from 19 to 74; 27% were female, 73% were male; 62% were Italian, 38% were Spanish. Not all patients had or experienced tinnitus at the time of the study.

The study proceeded as follows [46]:

– The evaluator explained the goals of the evaluation and established a friendly environment. Where required, the evaluator provided the users with guidelines on observational evaluation;
– For each task (T1, T2, T3)
  – The evaluator explained the goal of each task and asked the user to rate how easy/difficult he/she thinks the task is going to be (the EM);
  – The user performed the task;1As mentioned in Subsection 2.2.2, the acufenometry asks the user to reproduce a sound similar to that of his/her tinnitus via the app. Users who had never had tinnitus, were played samples of tinnitus sounds and were asked to match them with those on the application.
  – The evaluator asked the users to rate their experience of performing the task (the SEQ) and told them they could leave a comment on how to improve the application, if they wished.
– The user filled the SUS questionnaire.

The statistical analyses of these data was performed using R (version 3.4.1). The results are as follows:

###### 5.2.2.2.1 SUS analysis

Table 3 reports the average SUS score with confidence intervals, and the results of two Wilcoxon tests assessing whether the average SUS score is statistically higher than 50 and 70, which are commonly considered the thresholds for unacceptable/marginal/acceptable usability.

**Table 3:** SUS analysis.

Average with CI	*p*-value_>50_	*p*-value_>70_
73.6 [67.6, 79.6]	0.00004^*^	0.1982

The results show that the average app usability is 73.6, and – according to the tests, that is, the app’s usability is acceptable, above marginal but not above acceptable. Therefore, the SUS analysis shows that there is room for improvement. To understand where it is worth focusing, we proceed with the SEQ and EM analyses.

###### 5.2.2.2.2 SEQ analysis

**Figure 6:**
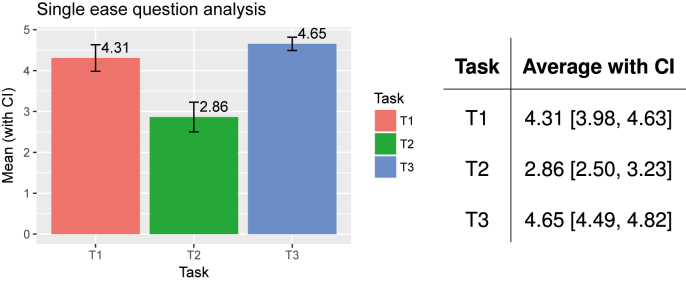
SEQ analysis. Audiometry (T1) and questionnaires (T3) are easy to perform. Acufenometry (T2) is difficult to perform.

Figure 6 summarises the results of the analysis. The bar chart on the left reports the average value of SEQ with confidence intervals, for each task. The table on the right details all numbers. The results show that the users found it easy to perform the audiometry and questionnaire tasks (T1 and T3, respectively), whereas the acufenometry (T2) was marked as a difficult task.

###### 5.2.2.2.3 EM analysis

**Figure 7:**
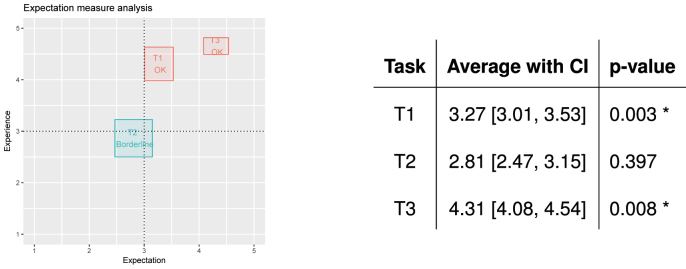
EM analysis, only acufenometry is located on the borderline position.

Figure 7 summarises the results of the analysis. The abscissa indicates the expectation, the ordinate indicates the experience (i.e., SEQ). The points and the related boxes contains the average expectation/experience, with confidence intervals for each task. The table on the right gives the numbers, with the result of a Wilcoxon test assessing whether the difference between the expectation and the experience of each task is statistically significant or not.

In relation to the graph, the tasks located in the different quadrants suggest different actions to researchers [46]:

– the upper/left quadrant contains tasks that were expected to be difficult but were found to be easy by users, i.e., tasks that are very well implemented and should be implemented;
– the lower/left quadrant contains tasks that were expected to be difficult and were experienced as such. They are the tasks that developers should re-implement in order to make their execution easier;
– the upper/right quadrant contains tasks that were both expected to be easy and were experienced as such, and therefore do not need to be changed;
– the lower/right quadrant contains tasks that were expected to be easy but users encountered difficulties while performing them, and thus must be prioritised by developers.

Borderline cases are also possible, i.e., tasks that are close to two (or more) quadrants. In these cases, confidence intervals may be used to provide a better understanding of the actions to be taken.

The results show that there is no need to make any changes/improvements to the audiometry and questionnaire tasks (T1 and T3, respectively) as they were found to be easier than expected. On the contrary, the level of difficulty of performing an acufenometry (T2) was in a borderline position; average values suggest that this task could be reimplementation in a different way to ease its performance.

## 6 Second iteration

The purpose of this section is to present the work carried out as part of a second iteration to improve the results obtained with the first iteration. More specifically, in this phase of the work, the efforts have been focused on improving all the issues related to the realisation of acufenometry, from the procedure implemented in the app (Section 6.1) up to the new evaluation (Section 6.2).

### 6.1 The app

**Figure 8:**
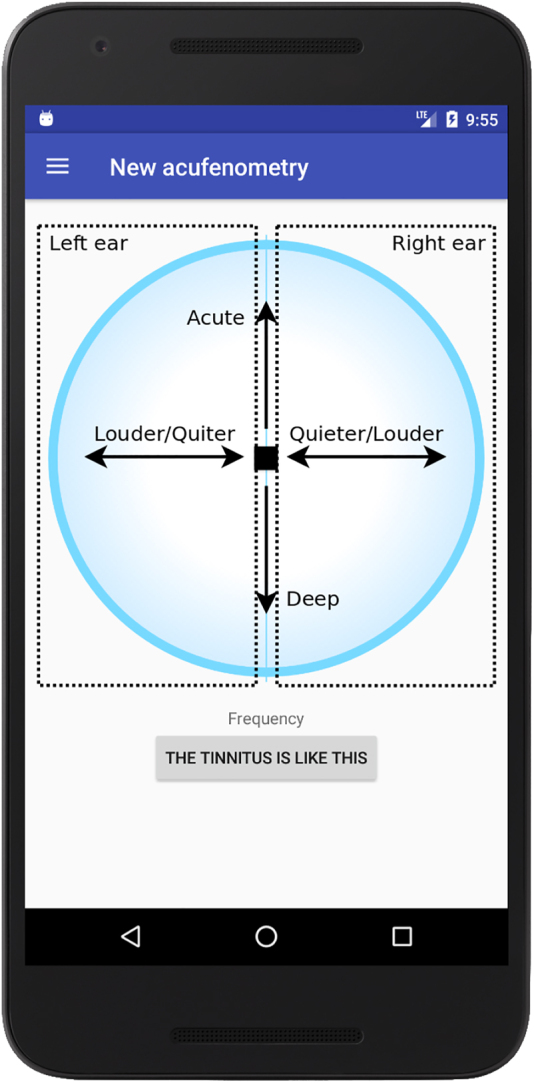
The improved interface for the acufenometry test.

Given the aforementioned results, the authors focused on the acufenometry task and improved the interface as shown in Figure 8. The interface was revised on the basis of the comments left by users: most of them reported that the spread of controls over the interface was confusing (see Figure 4). Therefore, in the new version of the interface, a user is only required to move the black dot: if the dot is on the left/right side of the circle, the sound is sent to the left/right ear; the closer the dot is to the top/bottom, the more acute/deep is the sound; the farther the dot from the centre, the louder the sound.

### 6.2 Evaluation

To evaluate the usability of the improved version of the app, the evaluators invited the same users who participated in the initial study, and proceeded as follows:

– The evaluator explained the goal of the new interface for acufenometry;
– The users performed the acufenometry;
– The evaluator asked the users to rate their experience of performing the acufenometry and to leave a comment, if possible, on how to improve the task;
– The users filled the SUS questionnaire.

The results presented below compare the experiences of users with the old/new acufenometry task and the old/new app.

**Figure 9:**
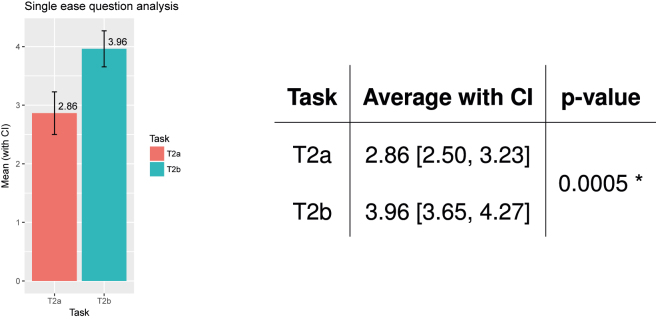
SEQ analysis, for acufenometry, old (T2a) and new (T2b) versions.

Firstly, Figure 9 summarises the results of the SEQ analysis. The bar chart on the left reports the average value of SEQ with confidence intervals, for the old (T2a) and the new version (T2b) of the acufenometry task. The table on the right reports all numbers, and the p-value of the Wilcoxon test used to assess whether the difference is statistically significant or not. The results confirm that the usability of the acufenometry task improved.

**Figure 10:**
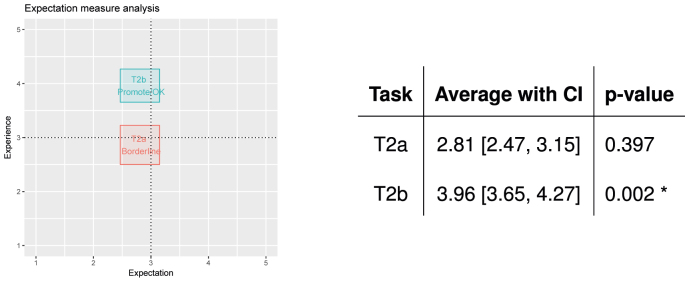
EM analysis for the acufenometry task, where the new version (T2b) overcomes the old version (T2a).

Then, Figure 10 depicts the results of the EM analysis, for both the old and new versions of the acufenometry task (T2a and T2b). The results show that now the acufenometry task (T2b) does not need improvements, it is easier than expected and can therefore help improve the app.

Table 4 contains the results of the SUS analysis, i.e., the average SUS score with confidence intervals for the new app version, as well as the results of the three Wilcoxon tests assessing whether the average SUS score is statistically higher than 50 and 70 (the already mentioned thresholds for an unacceptable/marginal/acceptable usability), and if the new app version improved in usability. The results show that the average app usability is 85.4, and – according to the tests – should offer more than an acceptable usability, and that this version is more useable than the previous one.

**Table 4:** SUS analysis.

Average with CI	*p*-value_>50_	*p*-value_>70_	*p*-value
85.4 [81.2, 89.6]	0.000004^*^	0.00005^*^	0.0003^*^

In conclusion, the novel implementation of the acufenometry task was perceived better than the initial one (from 2.86/5 to 3.96/5), and allowed to significantly improve the overall usability of the app (from 73.6/100 to 85.4/100).

## 7 Discussion

This paper presented a device and an app aimed at carrying out a clinical evaluation for a patient with tinnitus – through audiometry and acufenometry – as well as examining the impact of this condition on the patient’s quality of life – through questionnaires that look into the sleep quality, hyperacusis, and the evaluation of the disability. More concretely, the paper presented:

– automated audiometry reporting (see Figure 3), which was shown to be as accurate as the human reporting (Section 5.2.1);
– two successive implementations of the acufenometry task: in the second one, user experience was improved, of both the acufenometry task (see Figure 10) and the overall app usability (see Table 4);
– the automated scoring of questionnaires (see Figure 5).

It is worth highlighting the advance in the state of the art that this work implies, thanks to the creation of a new portable device that allows any user to perform self-assessments in a simple way, thanks to the capabilities and features offered by smartphones.

This fact makes the system better than the rest of the existing systems, from the point of view of information processing, allowing:

– The app’s communication interfaces offer great usability to patients, who can carry out the evaluation themselves, thus considerably facilitating the entire data collection process, without having to go to medical centres or needing experts to carry out the measurements;
– The use of a smartphone lowers the cost of the final device, because all the features they offer can be used and most patients already have their own mobile device. This increases the number of devices that can be purchased with the same budget, and thus makes it possible to reach a larger number of patients;
– Thanks to the connectivity of smartphones, information is highly available in multiple locations and can be used to be processed by more powerful computers and to perform more complex evaluations incorporating new information;
– Similarly, thanks to the ability to send the information (easily anonymized), it is possible to create a database with a large volume of patients that enriches the information available to draw new conclusions about tinnitus.

## 8 Conclusions

Given the results presented in the paper, the strengths of the app are twofold. First, the automated reporting feature, which – to the best of our knowledge – is the first attempt in this area. Second, good overall app usability and ease of performing the three tasks.

Our study has several limitations.

The first one regards the audiometric reporting procedure; it was tested without using the integrated headset. Although this shows that the algorithm works correctly, this does not guarantee that the audiometries produced by users will be perfectly reported, due to issues, e.g., in the wearing of the headset, the environment in which the test is performed. Despite the fact that the performed ergonomic study did not identify specific problems, as soon as the final device is developed, a further test will be performed to compare clinical reporting and automated reporting.

The second one regards the fact that the tinnitus matching procedure works only for tonal tinnitus. For pulsative tinnitus, the firmware device is not configured to emit a pulsatile tone yet, but its implementation is simple and straightforward. As for non-lateralized tinnitus, the patient is asked by the app to test the right ear, as of the common practice in the clinical settings.

The third one regards the fact that users with and without tinnitus were included in the sample. Given that there is a large variety of tinnitus patients, in terms of age, gender and type of tinnitus, creating a representative sample would have meant a very large number of subjects to be contacted [47]. On the other hand, it is almost ascertained that small samples in an iterative design process are more effective – in terms of cost-benefit analysis – to discover the most important usability issues [48, 49]. So, focusing on usability, and given the difficulty to enrol a representative sample of tinnitus patients, this choice was a necessary compromise.

The last and most important aspect when it comes to homologating the device and producing it for systematic use in medical centers is that the headset (including both the bone conduction and standard headphones) would need to be redesigned for industrial production, since the ones used in this work has been manually adapted from existing commercial products. Regarding the main hardware component and the app, they are easily replicable, so no changes would have to be made for industrial production.

In addition to facing the limitations previously described, the developments achieved under this framework give numerous opportunities for future research.

So far, the data produced by the app are stored in the user’s smartphone and can be shared, on a voluntary basis, with physicians or other users (by using the normal sharing techniques available on a common smartphone e.g., mail, bluetooth, messaging services). Nevertheless, the app may be used as a vector for creating a tinnitus data-center, which would store data on its diagnosis, evolution and management, these data may be statistically or automatically analysed, and therefore our knowledge of tinnitus may be greatly improved.

It should also be remarked that the research presented in this paper is the first part of our effort aimed at providing not only the tools necessary for diagnosing tinnitus – but also a set of personalised tools for its management, such as educational content [34, 50], masking [51, 52], distraction or neuromodulation [53]. Moreover, the information collected from the specific questionnaires helps find out whether or not the patient experiences hyperacusis or insomnia as a result of tinnitus: integrated with the type and degree of tinnitus, may give thorough and essential information for establishing a proper and personalised treatment. In the specific case of chronic tinnitus, cognitive behavioural therapy is currently the most accredited approach [54], sometimes associated with a pharmacological therapy. Furthermore, it is known that patients with decompensated chronic tinnitus experience high levels of stress and that the condition is closely associated with psychological disorders and high prevalence of anxiety, depression and hypochondriacal traits. Therefore, adding a specific section for psychological questionnaires and psychological interventions would be a further improvement in the future version of the app.

The app is available for smartphones and tablets running Android 5.0 and above, it is written in Java using Android Studio, and can be downloaded at the following link: http://vittorini.univaq.it/tinnitus/.
